# Bis(propane-1,3-diamine)­disaccharinatonickel(II)

**DOI:** 10.1107/S1600536812010525

**Published:** 2012-03-17

**Authors:** Gökhan Kaştaş, Can Kocabıyık

**Affiliations:** a Ondokuz Mayıs University, Faculty of Arts and Sciences, Department of Physics, 55139 Kurupelit Samsun, Turkey

## Abstract

In the title complex, [Ni(C_7_H_4_NO_3_S)_2_(C_3_H_10_N_2_)_2_] or [Ni(sac)_2_(pen)_2_] (sac = saccharinate or 1,1,3-trioxo-2,3-dihydro-1λ^6,2^-benzothia­zol-2-ide and pen = propane-1,3-diamine), the Ni^II^ ion sits on an inversion center, being coordinated by two N atoms of the sac ligands, which occupy *trans* positions, and four N atoms of the bidentate pen ligands to define a distorted octa­hedral geometry. The pen ligands chelate the metal ion, forming a six-membered ring which adopts a half-chair conformation, while the sac ligands adopt the most common coordination mode. The crystal packing is stabilized by N—H⋯O hydrogen bonds, which form a one-dimensional network along [010]. It is also supported by an N—H⋯S hydrogen bond between the amine group of the pen ligand and the sulfonyl group of the sac ligand.

## Related literature

For background to saccharin and the use of the saccharinato anion (sac), as a polyfunctional ligand, see: Baran & Yilmaz (2006 ▶); Heren *et al.* (2008 ▶); Paşaoğlu *et al.* (2007 ▶). For hydrogen-bond motifs, see: Bernstein *et al.* (1995 ▶). For a related structure, see: Bulut *et al.* (2007 ▶).
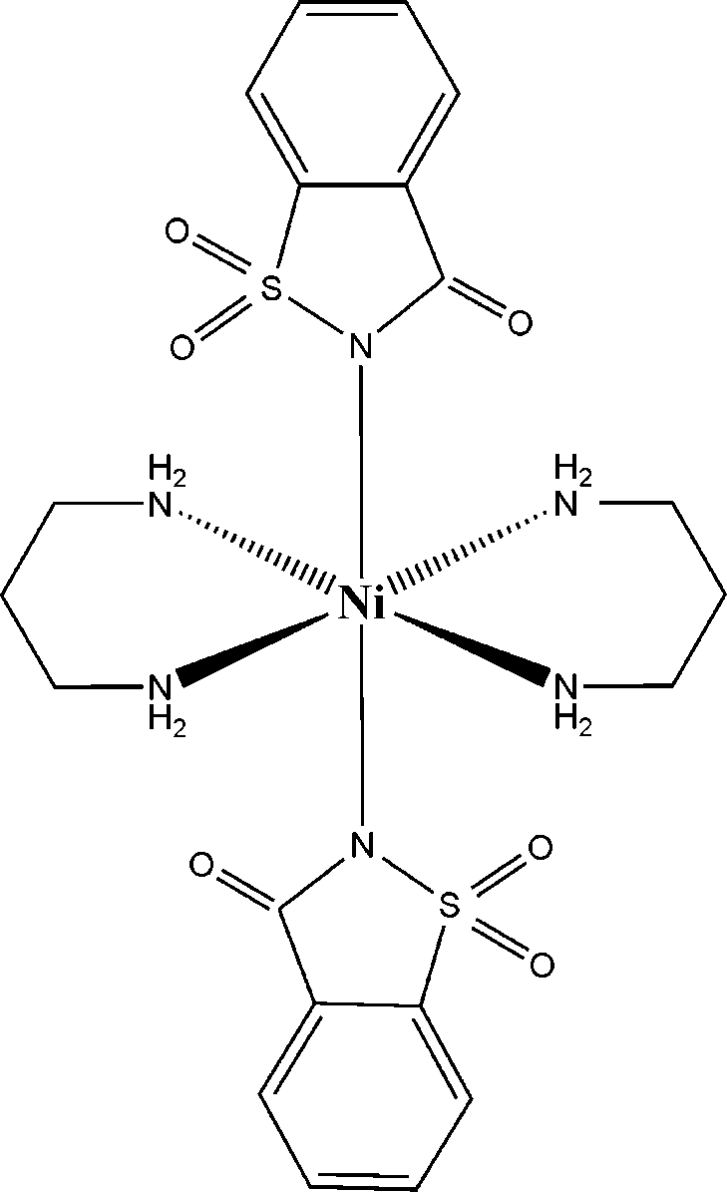


## Experimental

### 

#### Crystal data

[Ni(C_7_H_4_NO_3_S)_2_(C_3_H_10_N_2_)_2_]
*M* *_r_* = 571.31
Monoclinic, 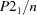

*a* = 11.447 (5) Å
*b* = 7.209 (6) Å
*c* = 15.236 (5) Å
β = 109.313 (5)°
*V* = 1186.5 (12) Å^3^
*Z* = 2
Mo *K*α radiation
μ = 1.04 mm^−1^
*T* = 296 K
0.52 × 0.35 × 0.15 mm

#### Data collection

Stoe IPDS 2 diffractometer
Absorption correction: integration (*X-RED*; Stoe & Cie, 2002 ▶) *T*_min_ = 0.673, *T*_max_ = 0.873
18160 measured reflections
2590 independent reflections
2334 reflections with *I* > 2σ(*I*)
*R*_int_ = 0.045

#### Refinement

*R*[*F* ^2^ > 2σ(*F* ^2^)] = 0.033
*wR*(*F* ^2^) = 0.101
*S* = 0.83
2590 reflections
177 parameters
H atoms treated by a mixture of independent and constrained refinement
Δρ_max_ = 0.74 e Å^−3^
Δρ_min_ = −0.37 e Å^−3^

### 

Data collection: *X-AREA* (Stoe & Cie, 2002 ▶); cell refinement: *X-AREA*; data reduction: *X-RED32* (Stoe & Cie, 2002 ▶); program(s) used to solve structure: *SHELXS97* (Sheldrick, 2008 ▶); program(s) used to refine structure: *SHELXL97* (Sheldrick, 2008 ▶); molecular graphics: *DIAMOND* (Brandenburg, 2006 ▶) and *Mercury* (Macrae *et al.*, 2006 ▶); software used to prepare material for publication: *WinGX* (Farrugia, 1999 ▶).

## Supplementary Material

Crystal structure: contains datablock(s) I, global. DOI: 10.1107/S1600536812010525/ds2179sup1.cif

Structure factors: contains datablock(s) I. DOI: 10.1107/S1600536812010525/ds2179Isup2.hkl

Additional supplementary materials:  crystallographic information; 3D view; checkCIF report

## Figures and Tables

**Table:** 

Ni1—N2	2.092 (2)
Ni1—N3	2.118 (2)
Ni1—N1	2.2604 (18)

**Table:** 

N2—Ni1—N3	91.43 (9)
N2—Ni1—N1	93.21 (8)
N3—Ni1—N1	88.87 (8)

**Table 2:** Hydrogen-bond geometry (Å, °)

*D*—H⋯*A*	*D*—H	H⋯*A*	*D*⋯*A*	*D*—H⋯*A*
N3—H3*A*⋯O3^i^	0.78 (3)	2.58 (3)	3.194 (3)	137 (3)
N3—H3*B*⋯O2^ii^	0.85 (4)	2.34 (4)	3.143 (3)	158 (3)
N3—H3*B*⋯S1^ii^	0.85 (4)	2.82 (4)	3.431 (2)	130 (3)
N2—H2*A*⋯O1^ii^	0.90 (4)	2.60 (3)	3.227 (3)	128 (3)
N2—H2*B*⋯O3^iii^	0.81 (4)	2.39 (4)	3.090 (3)	146 (3)
N3—H3*A*⋯O1	0.78 (3)	2.36 (3)	2.952 (3)	134 (3)
N2—H2*B*⋯O3	0.81 (4)	2.56 (4)	3.085 (3)	124 (3)

Symmetry codes: (i) 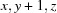; (ii) 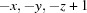; (iii) 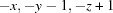.

**Fig. 1.**
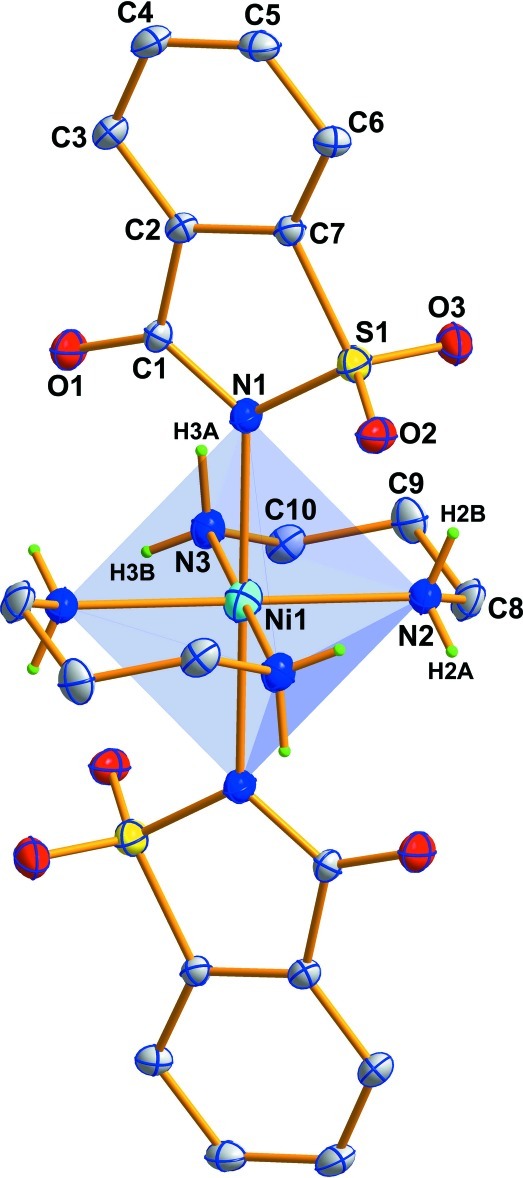
A molecular view of the complex, showing the atomic numbering scheme. Displacement ellipsoids are drawn at the 30% probability level. H atoms not involved in hydrogen bonding were omitted for clarity.

**Fig. 2.**
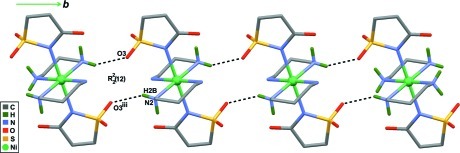
The centrosymmetric R22(12) rings connecting the discrete molecules along [010]. Benzene rings and some H atoms were omitted for clarity.

**Fig. 3.**
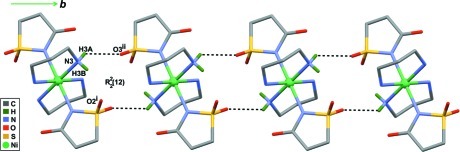
The formation of centrosymmetric R22(12) rings. Benzene rings and some H atoms were omitted for clarity.

## supplementary crystallographic information

### Comment

Saccharin (3*H*-benzisothiazol-3-one 1,1-dioxide or *o*-benzosulfimide) is a well known artificial sweetener. It is readily deprotonated to form saccharinato anion (sac), which is a versatile polyfunctional ligand (Baran & Yilmaz, 2006). Because of the biological significance of saccharin, there has been increased interest in its metal complexes, especially with first-row transition metals (Paşaoğlu *et al.*, 2007; Heren *et al.*, 2008. Saccharin, or its anion, may bond to metals by means of their imino nitrogen, carbonyl oxygen, or sulfonyl oxygen atoms. In the last two decades, the metal saccharinates and metal complexes including saccharin and various N-donor ligands (mono- or bidentate) have been intensively studied by many investigators. In the present study, the mixed-ligand Ni^II^ complex of saccharinate with pen (Scheme) is investigated.

In the title compound, Ni^II^ ion sits on an inversion center, being coordinated by two N atoms of sac ligand and four N atoms of bidentate pen ligands in *trans* positions. The bond distances and angles (Table 1) show that the coordination polyhedron of the Ni^II^ ion is a distorted octahedron. The equatorial plane of the octahedron is defined by the N atoms of pen ligands, whereas the axial positions are occupied by the N atoms of the sac ligands (Fig. 1). It is seen that the pen ligands chelate the metal ion to form a six-membered ring adopting a half-chair conformation while sac ligands prefer the most common coordination mode. The intra-ligand bond lengths and angles of the sac ligand are similar to those observed in previous studies (Paşaoğlu *et al.*, 2007; Heren *et al.*, 2008). The sac ligand is planar, with an r.m.s deviation of 0.022 Å. The dihedral angle between the equatorial plane and the mean plane of sac was measured as 84.50 (7)°. It is seen in Table 1 that the Ni—N_pen_ bond distances span the range 2.094 (3)–2.119 (3) Å, being shorter than Ni-Nsac distance (2.262 (2) Å) which is comparable to that observed in [Ni(C_7_H_4_NO_3_S)(C_5_H_9_N_3_)_2_] (2.2874 (19) Å) (Bulut *et al.*, 2007). The crystal packing of the complex is mainly stabilized by hydrogen bonds of N—H···O type (Table 2). Multiple H-bond donor and acceptor behaviors of amine and sulfonyl groups enable a variety of chain and ring systems in the crystal packing. For example, the inter-molecular N2—H2B···O3^iii^ (iii: -*x*, -1 - *y*, 1 - *z*) hydrogen bonds form centrosymmetric *R*^2^_2_(12) (Bernstein *et al.*, 1995) rings located at (0, 1/2+n, 1/2: n=0 or integer) positions to form one-dimensional hyrogen bonding network along [010] (Fig. 2). On the other hand, the same ring motifs are also formed by N3—H3B···O2^i^ (i:-*x*, -*y*, 1 - *z*) hydrogen bonds (Fig. 3).

### Experimental

An aquous solution of pen (2.54 mmol, 0.19 g) was added dropwise with stirring to a solution of [Ni(sac)_2_(H_2_O)_4_].2H_2_O (1.27 mmol, 1.00 g). The mixture was heated to 343 K in a temperature-controlled bath and stirred for 3 h. The green reaction mixture was filtered and left for crystallization in room temperature. After two weeks, the violet crystals of the title complex were selected for X-ray experiment. IR (KBr, ν cm^-1^), stretching vibrations: 3326–3290 (NH_2_), 3153–3060 (CH)_ring_, 2948–2881 (CH_2_) 1658 (C=O), 1575 (C=C)_sac_ 1406 (C=N)_pen_, 1325 (*CNS*)*_sym_*, 1269 (SO_2_)*_asym_* 1141 (SO_2_)*_sym_*, 950 (*CNS*)*_asym_*.

### Refinement

All H atoms except those involved in H-bonds were positioned geometrically and treated using a riding model, with the distances of 0.970 and 0.930 Å for methylene and aromatic groups, respectively. The displacement parameters of the H atoms were constrained with *U*_iso_(H) = 1.2*U*_eq_ for parent atoms.

### Crystal data

**Table:**

[Ni(C_7_H_4_NO_3_S)_2_(C_3_H_10_N_2_)_2_]	*F*(000) = 596
*M**_r_* = 571.31	*D*_x_ = 1.599 Mg m^−^^3^
Monoclinic, *P*2_1_/*n*	Mo *K*α radiation, λ = 0.71069 Å
Hall symbol: -P 2yn	Cell parameters from 27512 reflections
*a* = 11.447 (5) Å	θ = 1.9–28.1°
*b* = 7.209 (6) Å	µ = 1.04 mm^−^^1^
*c* = 15.236 (5) Å	*T* = 296 K
β = 109.313 (5)°	Prism, violet
*V* = 1186.5 (12) Å^3^	0.52 × 0.35 × 0.15 mm
*Z* = 2	

### Data collection

**Table:**

Stoe IPDS 2 diffractometer	2590 independent reflections
Radiation source: fine-focus sealed tube	2334 reflections with *I* > 2σ(*I*)
Graphite monochromator	*R*_int_ = 0.045
rotation method scans	θ_max_ = 27.0°, θ_min_ = 2.0°
Absorption correction: integration (*X-RED*; Stoe & Cie, 2002)	*h* = −14→14
*T*_min_ = 0.673, *T*_max_ = 0.873	*k* = −9→9
18160 measured reflections	*l* = −19→19

### Refinement

**Table:**

Refinement on *F*^2^	Secondary atom site location: difference Fourier map
Least-squares matrix: full	Hydrogen site location: inferred from neighbouring sites
*R*[*F*^2^ > 2σ(*F*^2^)] = 0.033	H atoms treated by a mixture of independent and constrained refinement
*wR*(*F*^2^) = 0.101	*w* = 1/[σ^2^(*F*_o_^2^) + (0.0792*P*)^2^ + 0.9405*P*] where *P* = (*F*_o_^2^ + 2*F*_c_^2^)/3
*S* = 0.83	(Δ/σ)_max_ = 0.001
2590 reflections	Δρ_max_ = 0.74 e Å^−^^3^
177 parameters	Δρ_min_ = −0.37 e Å^−^^3^
0 restraints	Extinction correction: *SHELXL97* (Sheldrick, 2008), Fc^*^=kFc[1+0.001xFc^2^λ^3^/sin(2θ)]^-1/4^
Primary atom site location: structure-invariant direct methods	Extinction coefficient: 0.0040 (12)

### Special details

**Table:**

Geometry. All e.s.d.'s (except the e.s.d. in the dihedral angle between two l.s. planes) are estimated using the full covariance matrix. The cell e.s.d.'s are taken into account individually in the estimation of e.s.d.'s in distances, angles and torsion angles; correlations between e.s.d.'s in cell parameters are only used when they are defined by crystal symmetry. An approximate (isotropic) treatment of cell e.s.d.'s is used for estimating e.s.d.'s involving l.s. planes.
Refinement. Refinement of *F*^2^ against ALL reflections. The weighted *R*-factor *wR* and goodness of fit *S* are based on *F*^2^, conventional *R*-factors *R* are based on *F*, with *F* set to zero for negative *F*^2^. The threshold expression of *F*^2^ > σ(*F*^2^) is used only for calculating *R*-factors(gt) *etc*. and is not relevant to the choice of reflections for refinement. *R*-factors based on *F*^2^ are statistically about twice as large as those based on *F*, and *R*- factors based on ALL data will be even larger.

### Fractional atomic coordinates and isotropic or equivalent isotropic displacement parameters (Å^2^)

**Table:**

	*x*	*y*	*z*	*U*_iso_*/*U*_eq_	
Ni1	0.0000	0.0000	0.5000	0.03195 (14)	
S1	−0.09614 (5)	−0.33212 (7)	0.62118 (4)	0.03795 (16)	
O3	−0.00193 (18)	−0.4563 (2)	0.61249 (13)	0.0506 (4)	
N1	−0.05429 (17)	−0.1179 (2)	0.61849 (12)	0.0384 (4)	
O2	−0.21766 (18)	−0.3644 (3)	0.55571 (12)	0.0567 (5)	
N3	0.1349 (2)	0.1650 (3)	0.59749 (14)	0.0408 (4)	
C2	−0.0733 (2)	−0.1541 (3)	0.76883 (15)	0.0409 (5)	
O1	−0.02673 (19)	0.1378 (2)	0.71301 (14)	0.0585 (5)	
C7	−0.10157 (19)	−0.3318 (3)	0.73503 (15)	0.0378 (4)	
C1	−0.0490 (2)	−0.0281 (3)	0.69877 (17)	0.0414 (5)	
N2	0.12960 (19)	−0.2097 (3)	0.50843 (14)	0.0416 (4)	
C6	−0.1277 (2)	−0.4744 (4)	0.78602 (17)	0.0459 (5)	
H6	−0.1467	−0.5931	0.7616	0.055*	
C5	−0.1241 (3)	−0.4313 (5)	0.87586 (19)	0.0578 (7)	
H5	−0.1404	−0.5231	0.9131	0.069*	
C3	−0.0707 (3)	−0.1121 (4)	0.85797 (18)	0.0566 (6)	
H3	−0.0522	0.0071	0.8819	0.068*	
C9	0.3068 (2)	−0.0680 (5)	0.6283 (2)	0.0633 (7)	
H9A	0.3305	−0.0113	0.5790	0.076*	
H9B	0.3824	−0.1027	0.6773	0.076*	
C8	0.2373 (2)	−0.2388 (4)	0.59136 (17)	0.0527 (6)	
H8A	0.2924	−0.3264	0.5767	0.063*	
H8B	0.2100	−0.2939	0.6394	0.063*	
C4	−0.0965 (3)	−0.2530 (5)	0.91060 (19)	0.0641 (8)	
H4	−0.0952	−0.2274	0.9707	0.077*	
C10	0.2423 (2)	0.0754 (5)	0.66625 (18)	0.0614 (7)	
H10A	0.2148	0.0179	0.7136	0.074*	
H10B	0.3018	0.1708	0.6964	0.074*	
H3A	0.098 (3)	0.220 (4)	0.623 (2)	0.045 (7)*	
H3B	0.168 (3)	0.238 (5)	0.569 (2)	0.079 (11)*	
H2A	0.157 (3)	−0.179 (5)	0.461 (2)	0.068 (9)*	
H2B	0.091 (3)	−0.306 (5)	0.498 (2)	0.071 (10)*	

### Atomic displacement parameters (Å^2^)

**Table:**

	*U*^11^	*U*^22^	*U*^33^	*U*^12^	*U*^13^	*U*^23^
Ni1	0.0364 (2)	0.0281 (2)	0.0298 (2)	−0.00006 (13)	0.00887 (14)	−0.00239 (12)
S1	0.0495 (3)	0.0318 (3)	0.0353 (3)	−0.0060 (2)	0.0177 (2)	−0.00277 (19)
O3	0.0724 (12)	0.0318 (8)	0.0610 (11)	−0.0004 (8)	0.0399 (9)	−0.0036 (7)
N1	0.0512 (10)	0.0299 (8)	0.0373 (9)	−0.0036 (7)	0.0189 (8)	−0.0019 (7)
O2	0.0613 (10)	0.0609 (11)	0.0423 (9)	−0.0209 (9)	0.0098 (8)	−0.0012 (8)
N3	0.0475 (10)	0.0392 (10)	0.0351 (9)	−0.0075 (8)	0.0125 (8)	−0.0044 (8)
C2	0.0426 (11)	0.0438 (12)	0.0393 (11)	−0.0010 (9)	0.0176 (9)	−0.0056 (9)
O1	0.0853 (13)	0.0364 (9)	0.0662 (12)	−0.0125 (9)	0.0419 (11)	−0.0154 (8)
C7	0.0375 (10)	0.0408 (11)	0.0371 (10)	0.0010 (8)	0.0152 (8)	−0.0012 (8)
C1	0.0471 (11)	0.0362 (11)	0.0448 (12)	−0.0018 (9)	0.0204 (10)	−0.0074 (9)
N2	0.0458 (10)	0.0352 (10)	0.0430 (10)	0.0030 (8)	0.0138 (8)	−0.0004 (8)
C6	0.0488 (12)	0.0479 (13)	0.0453 (12)	0.0005 (10)	0.0214 (10)	0.0061 (10)
C5	0.0595 (15)	0.0734 (18)	0.0474 (13)	−0.0001 (13)	0.0270 (12)	0.0104 (13)
C3	0.0673 (16)	0.0627 (16)	0.0461 (13)	−0.0074 (13)	0.0275 (12)	−0.0155 (12)
C9	0.0435 (13)	0.0687 (18)	0.0675 (17)	0.0012 (12)	0.0045 (12)	−0.0007 (15)
C8	0.0532 (13)	0.0592 (15)	0.0434 (12)	0.0150 (11)	0.0128 (10)	0.0037 (11)
C4	0.0736 (18)	0.086 (2)	0.0411 (13)	−0.0076 (16)	0.0303 (13)	−0.0077 (13)
C10	0.0522 (14)	0.081 (2)	0.0412 (13)	−0.0014 (14)	0.0020 (11)	−0.0180 (13)

### Geometric parameters (Å, º)

**Table:**

Ni1—N2	2.092 (2)	N2—C8	1.459 (3)
Ni1—N2^i^	2.092 (2)	N2—H2A	0.90 (4)
Ni1—N3	2.118 (2)	N2—H2B	0.81 (4)
Ni1—N3^i^	2.118 (2)	C6—C5	1.391 (4)
Ni1—N1^i^	2.2604 (18)	C6—H6	0.9300
Ni1—N1	2.2604 (18)	C5—C4	1.386 (5)
S1—O2	1.4375 (19)	C5—H5	0.9300
S1—O3	1.4409 (18)	C3—C4	1.385 (4)
S1—N1	1.622 (2)	C3—H3	0.9300
S1—C7	1.756 (2)	C9—C8	1.473 (4)
N1—C1	1.367 (3)	C9—C10	1.493 (4)
N3—C10	1.475 (3)	C9—H9A	0.9700
N3—H3A	0.78 (3)	C9—H9B	0.9700
N3—H3B	0.85 (4)	C8—H8A	0.9700
C2—C7	1.378 (3)	C8—H8B	0.9700
C2—C3	1.382 (3)	C4—H4	0.9300
C2—C1	1.497 (3)	C10—H10A	0.9700
O1—C1	1.227 (3)	C10—H10B	0.9700
C7—C6	1.379 (3)		
			
N2—Ni1—N2^i^	180.00 (12)	N1—C1—C2	112.75 (19)
N2—Ni1—N3	91.43 (9)	C8—N2—Ni1	122.38 (16)
N2^i^—Ni1—N3	88.57 (9)	C8—N2—H2A	108 (2)
N2—Ni1—N3^i^	88.57 (9)	Ni1—N2—H2A	101 (2)
N2^i^—Ni1—N3^i^	91.43 (9)	C8—N2—H2B	107 (3)
N3—Ni1—N3^i^	180.0	Ni1—N2—H2B	106 (2)
N2—Ni1—N1^i^	86.79 (8)	H2A—N2—H2B	112 (3)
N2^i^—Ni1—N1^i^	93.21 (8)	C7—C6—C5	116.5 (2)
N3—Ni1—N1^i^	91.13 (8)	C7—C6—H6	121.7
N3^i^—Ni1—N1^i^	88.87 (8)	C5—C6—H6	121.7
N2—Ni1—N1	93.21 (8)	C4—C5—C6	120.8 (3)
N2^i^—Ni1—N1	86.79 (8)	C4—C5—H5	119.6
N3—Ni1—N1	88.87 (8)	C6—C5—H5	119.6
N3^i^—Ni1—N1	91.13 (8)	C2—C3—C4	117.9 (3)
N1^i^—Ni1—N1	180.000 (1)	C2—C3—H3	121.0
O2—S1—O3	114.68 (12)	C4—C3—H3	121.0
O2—S1—N1	111.28 (11)	C8—C9—C10	116.9 (2)
O3—S1—N1	110.67 (11)	C8—C9—H9A	108.1
O2—S1—C7	110.08 (11)	C10—C9—H9A	108.1
O3—S1—C7	111.32 (11)	C8—C9—H9B	108.1
N1—S1—C7	97.53 (10)	C10—C9—H9B	108.1
C1—N1—S1	110.76 (15)	H9A—C9—H9B	107.3
C1—N1—Ni1	126.42 (15)	N2—C8—C9	113.9 (2)
S1—N1—Ni1	122.67 (9)	N2—C8—H8A	108.8
C10—N3—Ni1	119.70 (17)	C9—C8—H8A	108.8
C10—N3—H3A	109 (2)	N2—C8—H8B	108.8
Ni1—N3—H3A	105 (2)	C9—C8—H8B	108.8
C10—N3—H3B	103 (2)	H8A—C8—H8B	107.7
Ni1—N3—H3B	110 (2)	C3—C4—C5	121.6 (2)
H3A—N3—H3B	110 (3)	C3—C4—H4	119.2
C7—C2—C3	119.8 (2)	C5—C4—H4	119.2
C7—C2—C1	111.95 (19)	N3—C10—C9	115.5 (2)
C3—C2—C1	128.2 (2)	N3—C10—H10A	108.4
C2—C7—C6	123.3 (2)	C9—C10—H10A	108.4
C2—C7—S1	106.84 (16)	N3—C10—H10B	108.4
C6—C7—S1	129.82 (18)	C9—C10—H10B	108.4
O1—C1—N1	124.4 (2)	H10A—C10—H10B	107.5
O1—C1—C2	122.9 (2)		
			
O2—S1—N1—C1	111.22 (18)	O3—S1—C7—C6	−62.0 (2)
O3—S1—N1—C1	−120.04 (17)	N1—S1—C7—C6	−177.7 (2)
C7—S1—N1—C1	−3.81 (18)	S1—N1—C1—O1	−175.6 (2)
O2—S1—N1—Ni1	−72.90 (14)	Ni1—N1—C1—O1	8.7 (4)
O3—S1—N1—Ni1	55.84 (15)	S1—N1—C1—C2	4.4 (2)
C7—S1—N1—Ni1	172.06 (11)	Ni1—N1—C1—C2	−171.32 (14)
N2—Ni1—N1—C1	122.6 (2)	C7—C2—C1—O1	177.2 (2)
N2^i^—Ni1—N1—C1	−57.4 (2)	C3—C2—C1—O1	−2.7 (4)
N3—Ni1—N1—C1	31.3 (2)	C7—C2—C1—N1	−2.8 (3)
N3^i^—Ni1—N1—C1	−148.7 (2)	C3—C2—C1—N1	177.3 (2)
N2—Ni1—N1—S1	−52.59 (13)	N3—Ni1—N2—C8	27.2 (2)
N2^i^—Ni1—N1—S1	127.41 (13)	N3^i^—Ni1—N2—C8	−152.8 (2)
N3—Ni1—N1—S1	−143.95 (13)	N1^i^—Ni1—N2—C8	118.2 (2)
N3^i^—Ni1—N1—S1	36.05 (13)	N1—Ni1—N2—C8	−61.8 (2)
N2—Ni1—N3—C10	−26.0 (2)	C2—C7—C6—C5	−0.2 (4)
N2^i^—Ni1—N3—C10	154.0 (2)	S1—C7—C6—C5	179.64 (19)
N1^i^—Ni1—N3—C10	−112.9 (2)	C7—C6—C5—C4	0.5 (4)
N1—Ni1—N3—C10	67.1 (2)	C7—C2—C3—C4	0.3 (4)
C3—C2—C7—C6	−0.2 (4)	C1—C2—C3—C4	−179.9 (3)
C1—C2—C7—C6	179.9 (2)	Ni1—N2—C8—C9	−48.1 (3)
C3—C2—C7—S1	179.9 (2)	C10—C9—C8—N2	66.0 (3)
C1—C2—C7—S1	0.1 (2)	C2—C3—C4—C5	0.0 (5)
O2—S1—C7—C2	−113.86 (17)	C6—C5—C4—C3	−0.4 (5)
O3—S1—C7—C2	117.82 (17)	Ni1—N3—C10—C9	47.0 (3)
N1—S1—C7—C2	2.12 (17)	C8—C9—C10—N3	−66.7 (4)
O2—S1—C7—C6	66.3 (2)		

Symmetry code: (i) −*x*, −*y*, −*z*+1.

### Hydrogen-bond geometry (Å, º)

**Table:**

*D*—H···*A*	*D*—H	H···*A*	*D*···*A*	*D*—H···*A*
N3—H3*A*···O3^ii^	0.78 (3)	2.58 (3)	3.194 (3)	137 (3)
N3—H3*B*···O2^i^	0.85 (4)	2.34 (4)	3.143 (3)	158 (3)
N3—H3*B*···S1^i^	0.85 (4)	2.82 (4)	3.431 (2)	130 (3)
N2—H2*A*···O1^i^	0.90 (4)	2.60 (3)	3.227 (3)	128 (3)
N2—H2*B*···O3^iii^	0.81 (4)	2.39 (4)	3.090 (3)	146 (3)
N3—H3*A*···O1	0.78 (3)	2.36 (3)	2.952 (3)	134 (3)
N2—H2*B*···O3	0.81 (4)	2.56 (4)	3.085 (3)	124 (3)

Symmetry codes: (i) −*x*, −*y*, −*z*+1; (ii) *x*, *y*+1, *z*; (iii) −*x*, −*y*−1, −*z*+1.
